# Neurorehabilitation and white matter repair in traumatic spinal cord injury: a dialogue between clinical and preclinical studies

**DOI:** 10.3389/fneur.2025.1532056

**Published:** 2025-06-18

**Authors:** Vito Antonio Baldassarro, Ilaria Baroncini, Laura Calzà, Francesca Ciardulli, Luca Lorenzini, Francesco Giuseppe Materazzi, Francesca Merighi, Corinne Quadalti, Lucia Ricci, Francesca Serafino, Laura Simoncini

**Affiliations:** ^1^Department of Veterinary Medical Sciences (DIMEVET), University of Bologna, Bologna, Italy; ^2^Montecatone Rehabilitation Institute, Imola (Bologna), Italy; ^3^Department of Pharmacy and Biotechnologies (FaBiT), University of Bologna, Bologna, Italy; ^4^Interdepartmental Center for Industrial Research, Life Science and Health Technologies (CIRI-SdV), University of Bologna, Bologna, Italy

**Keywords:** white matter (WM), oligodendrocyte precursor cells (OPCs), regenerative neurorehabilitation, spinal cord injury, myelin regeneration

## Abstract

The central nervous system (CNS) has very limited repair capabilities, and the functional adaptation/compensation after acute injuries is attributed to the significant plasticity of neural circuits, in particular at the synaptic level. However, neurons are only one of the cellular components of the CNS, with gray matter (GM) comprising around 50% of its structure, compared to white matter (WM), where oligodendrocytes (Ols) form the myelin sheath and ensure the isolation of axons for proper electrical conductivity elicited by action potentials. WM is characterized by two remarkable properties: myelin plasticity, defined as experience-induced changes in myelination that mediate long-lasting changes in neural circuit function, and myelin repair, which can be complete and functionally effective and represents the CNS's only true reparative capability. Oligodendrocyte precursor cells (OPCs), accounting for 5–8% of the total CNS cells, are responsible for myelin plasticity and repair. OPCs are generated during development, are widely distributed across both white and gray matter, and remain quiescent until appropriate stimuli, such as functional requests or injuries, arise. Under these conditions, endogenous OPCs, as well as new OPCs derived from the proliferation and differentiation of endogenous neural stem cells, migrate toward axons and differentiate into mature OLs capable of wrapping axons and forming the myelin sheaths. In this review article, we discuss WM plasticity and myelin repair through OPC-dependent endogenous regeneration within the context of spinal cord injury (SCI) and related neurorehabilitation approaches. Clinical data, such as imaging information, pertain to changes in WM during various phases of SCI and have been collected in different rehabilitation contexts. Preclinical data focus on physical stimuli that can enhance the myelin repair capacity of OPCs within the context of the oligo-axon unit. The potential role of myelin regeneration by endogenous stem/precursor cells is finally discussed in the context of regenerative neurorehabilitation for SCI.

## 1 Regenerative rehabilitation and endogenous stem and precursor cells

The dialogue between preclinical and clinical research is of outstanding importance to accelerate the bench-to-bed pathway and to better focus on targets and strategies of preclinical translational research (1, 2). While this general (and obvious) concept is often kept under wraps and poorly practiced, regenerative rehabilitation has brought it back into the research methodology spotlight. Regenerative rehabilitation has been proposed as the optimized application of rehabilitation science to promote regenerative therapies (3). It combines a preeminent research topic, i.e., regenerative medicine, and a prominent clinical field, i.e., rehabilitation science, with the specific aim of highlighting how these two approaches can mutually enhance each other, synergizing their respective contributions to improving clinical outcomes after an injury, disorder, or degeneration (4).

While originally focused on the musculoskeletal system, regenerative rehabilitation is now directed toward neuronal functional recovery, given the significant effort in cellular therapies for neurological conditions. Spinal cord injury (SCI) is one of the neurological disorders where regenerative rehabilitation can be applied. SCI is a very severe condition with various motor, sensory, and autonomic sequelae, and it has an enormous impact on the psychosocial aspects of patients' lives. Its incidence generally exhibits two peaks: one in individuals younger than 30 years (mainly traumatic SCI due to traffic or sports accidents, and falls) and another in those older than 60 years (mainly non-traumatic SCI, due to vascular injury, spine osteoarthritis, tumors, etc.). The overall global incidence of traumatic SCI is estimated to be 10.5 cases per 100,000 persons. The average male/female (M/F) ratio is 3.37 worldwide, and the mean age of patients with traumatic SCI is 39.8 (SD ± 12.2) (5). SCI is classified as an “orphan disorder” (ORPHA:90,058), and therapies are limited to spinal column stabilization and decompression surgery in the acute phase, followed by rehabilitative treatments.

SCI offers a unique opportunity to address the complex interactions between disciplines, as required by regenerative therapies. It is one of the neurological conditions primarily explored for regenerative medicine at the preclinical level, including cell therapies and biomaterials, and a condition in which clinical rehabilitation is the only true possibility to improve functional outcomes. From a neurobiological perspective, traumatic SCI presents the unique scenario of a sudden injury in otherwise healthy tissue. Moreover, the epidemiology of traumatic SCI indicates a high prevalence among young, male subjects, creating a relatively homogeneous incidence profile compared to other conditions.

Rehabilitation approaches include mechanical stimuli, such as rehabilitative training, tissue loading, stretching, joint mobilization, and traction; as well as physical stimuli, including electrical and magnetic stimulation, temperature gradients, and ultrasound stimulation. Regenerative therapies are based on cellular therapies and related supports (biomaterials, drugs, bioactive molecules), which include not only cell transplantation but also the mobilization of endogenous stem cells and precursors.

The presence of stem cells and undifferentiated precursors in the mature CNS is well established in many mammalian species, including humans. Adult neural stem cells (aNSCs) are localized in histologically defined microdomains referred to as “niches” in specific brain areas, such as the subventricular zone of the lateral ventricles and the subgranular zone of the dentate gyrus. NSCs contribute to brain function through adult neurogenesis directed toward specific neuron types and in injury responses, such as those following stroke or trauma (6). NSCs are also present in the ependymal region of the central canal of the adult mammalian spinal cord (7).

Moreover, a large population of undifferentiated oligodendrocyte progenitors, known as oligodendrocyte precursor cells (OPCs), populates the entire adult CNS (8). In rats, OPCs account for ~2–3% of total cells in gray matter (GM) and 5–8% in white matter (WM), while in humans, quantitative stereological analysis of the corpus callosum records ~260 cells/mm^2^ in newborns, which is reduced during early development and stabilizes at a level of about 160 cells/mm^2^ at ~5 years of age (9). OPCs are responsible for myelin turnover and myelin repair in the adult brain, although recent studies indicate that these cells have multiple physiological roles based on synaptic contacts with axons and electrical activity (8). Both stem and precursor cells are regarded as potential intervention targets in neurological diseases and lesions (10–12).

In this review, we will discuss the impact of rehabilitation on myelin repair through the mobilization, migration, and differentiation of endogenous stem and precursor cells into myelinating oligodendrocytes (OLs), based on both preclinical and clinical data. Preclinical data will provide evidence of myelin plasticity and repair involving endogenous cells, including the influence of mechanical signals; clinical data will describe white matter post-injury changes, considering different rehabilitation approaches. Exogenous cell therapies for neuroinflammation control and myelin repair are outside the scope of this review. Several comprehensive review articles on both preclinical and clinical cell transplantation studies have been recently published (13–16).

## 2 A common preclinical and clinic ground: myelin dynamics across lifespan

### 2.1 Developmental myelination

WM consists largely of bundled nerve fibers, formed by axons surrounded by a dense, insulating sheath of myelin. This combined structure allows for the propagation of saltatory impulses and the conduction of signals along the axons, together with protection and metabolic support for neurons within a complex histological microdomain that includes axons, oligodendrocytes, as well as astrocytes and other cell types (17, 18). Myelinated axons conduct impulses many times faster than unmyelinated ones of the same cross-sectional size and are less vulnerable than “nude” axons.

In general, myelination is a postnatal process in experimental rodents and in humans, both of which are born with a virtually unmyelinated CNS. Myelin is formed by OLs, which differentiate from OPCs through a complex process that culminates in the wrapping of several axons by a single OL in concentric layers of plasma membrane that compacts, expelling cytoplasm to form internodes. Each of these layers results in a periodicity of ~12 nm. Internodal segments alternate with nodes of Ranvier (unwrapped axonal membrane, with a ~1-μm gap between adjacent myelin internodes), where an accumulation of voltage-gated sodium channels allows for action potential propagation via saltatory conduction (19).

Developmental myelination follows a specific spatiotemporal pattern, varying widely across brain regions, and correlating with the emergence, maturation, and maintenance of proper function in circuits supporting cognitive, somatosensory, and motor processes. In general, the timing of myelination and functional maturation are closely correlated, starting in CNS areas dedicated to basic homeostasis, proceeding to regions controlling more complex tasks, and finally arriving at areas involved in higher intellectual functions. Developmental myelination is particularly protracted in humans and continues at least into the fourth decade of life.

OLs differentiate from OPCs, which are generated from NSCs in the ventricular zone of the neural tube. In the spinal cord, these precursors are located in the same regional domain that gives rise to motor neurons (20). OPCs are unipotent progenitor cells that can divide either symmetrically to produce two daughter OPCs or asymmetrically to self-renew while producing one daughter OL. OPCs proliferate and migrate, populating the entire developing CNS, so that also the OL population expands dramatically following birth (21).

Migration is regulated by several molecular cues that promote cellular motility, including growth factors, neurotransmitters, chemokines, and chemoattractants or chemorepellents, such as semaphorins and netrins, which also direct long-range migration (20). Moreover, recent evidence shows that OPCs migrate through the developing CNS along the vasculature, which appears before the emergence of OPCs. In the developing spinal cord, OPCs use vasculature as a scaffold, “crawling” or “inchworming” along the abluminal endothelial surface and “jumping” between vessels. The vasculature-released Wnt signals seem to regulate the distance of OPC migration from the vessels into the surrounding parenchyma (22). Notably, in the postnatal CNS, OPCs direct angiogenesis to meet the energy requirements for differentiation and myelination via the OPC-intrinsic hypoxia-inducible factor (HIF) molecular pathway (23), and this mechanism appears to be significant in promoting and directing remyelination after injuries (24).

During migration, some OPCs differentiate into OLs, becoming morphologically more complex and functionally less prone to continue migrating, especially under the influence of local signals that drive differentiation. An important stop signal in the developing spinal cord seems to be the transient expression of the chemokine CXCL1 in a subset of astrocytes in the WM; several components of the extracellular matrix, such as tenascin-C, are also involved. Notably, site-specific differences in ECM composition could contribute to regional heterogeneity of OPC/OL subpopulations.

The maturation of OPCs into myelinating OLs is a highly orchestrated and complex process that requires exposure to a combination of growth factors, hormonal influences, and epigenetic modifications. Specific soluble signals drive OPCs out of the cell cycle toward mature OLs that express myelin proteins, including myelin basic protein (MBP) and proteolipid protein (PLP) (25, 26). Notably, OPCs receive excitatory glutamatergic input from neurons, and the surface expression of the glutamatergic AMPA receptor changes throughout the OL lineage, contributing to OPC/OL heterogeneity in the mature CNS. Similarly, almost all GABA receptor subtypes are expressed in oligodendroglial cells, allowing for neuron-oligo communication (27–29).

Axons play key roles in OPC terminal differentiation and proper OL organization by expressing growth factors and cell adhesion molecules that guide the timing and extent of myelination (30). Moreover, it is now clear that electrical activity promotes myelination by increasing OPC proliferation, myelin formation, and the thickness of the myelin sheath. Axons influence the number of myelin wraps and help to temporally and spatially regulate the myelination process, ensuring that neural circuit structures develop in a coordinated manner with the functional maturation related to axon myelination.

Regional heterogeneity is a key feature of OL biology and related myelin formation strategies, being directly related to the regional origin during the three migratory waves that guide the OPC population of the GM and WM during development. For example, OLs in the mouse cerebral cortex make shorter internodes than their counterparts in the corpus callosum, subcortical GM, brainstem, or spinal cord (31). Moreover, the WM microenvironment more than the GM favors proliferation and the constant generation of mature, myelinating OLs from OPCs (32), and OPCs in the WM, but not in the GM, are responsive to the mitogen platelet-derived growth factor (PDGF) (33). At the end, six mature OL molecular states across the CNS have been recognized by single-cell transcriptomics, possibly due to the different microenvironments to which OPCs are exposed during differentiation (34).

Depending on the CNS region, each OL will myelinate between 20 and 60 axons, with myelin internodes ranging from 20 to 200 μm in length (31). Usually, mature OLs in the mammalian brain myelinate axons with diameters >0.2 μm, but unmyelinated axons with diameters between 0.2 μm and 0.8 μm can also be found. Myelin *in situ* has a water content of about 40%. In contrast to most biological membranes, the dry mass is composed of a high proportion of lipids (70 to 85%) and a low proportion of proteins (15 to 30%), such as PLP, MBP, 2′:3′-cyclic nucleotide-3′-phosphodiesterase (CNPase), myelin-associated glycoprotein (MAG), and other glycoproteins (35).

### 2.2 White matter microstructure: the neuron–oligodendrocyte unit

*In vitro* studies based on animal and human cells have shown that OLs possess an intrinsic capacity for myelination of any natural or artificial fiber of the correct size, such as paraformaldehyde-fixed axons and polymeric filaments (36, 37). However, only axons, and not glial cell processes, vascular structures, neuronal dendrites, or cell bodies, become myelinated *in situ*, and appropriately sized fibers can be unmyelinated *in vivo*. This means that an active role of appropriately sized axons is needed to properly refine the innate myelination program of OPC/OL, suggesting that neurons instruct their own myelination. Although the molecular mechanisms supporting this interaction are still far from being elucidated, surface-localized cell adhesion molecules, signaling molecules, and receptors are reliable candidates.

Moreover, recent studies indicated that many, though not all, OPCs are electrically excitable, and some OPCs are even able to generate action potentials (8). This is related to the expression of several types of voltage-gated channels, including fast tetrodotoxin (TTX)-sensitive Na+ channels, low-voltage- and high-voltage-activated Ca2+channels (T- and L-types, or Cav1.2, Cav1.3, Cav3.1, and Cav3.2), and various types of K+ channels on the cell membrane. Moreover, fully operational synapses capable of vesicular exocytosis of neurotransmitters, quantal responses, facilitation, depression, and presynaptic inhibition are formed by neuronal terminals on the processes of OPCs (38). This provides a direct link between neural activity and OPC growth and differentiation.

More recently, RNA-seq analyses in OPCs revealed the expression of several other synaptic adhesion proteins, including Lrrtm1 and Lrrtm2, Neuroligin 1, Neuroligin 2, Cadm1a, and Cadm1b (39).

It is now recognized that direct synapses exist from neurons to the myelin sheath, as suggested by the accumulation of presynaptic vesicle machinery on axons beneath myelin sheaths, and by the expression of post-synaptic protein PSD-95 in myelinating OL at multiple locations on the myelin sheath. Moreover, in *in vivo* experiments using the zebrafish model of myelination, the fusion of synaptic vesicles has been observed, indicating this classical synaptic molecular mechanism between neurons and OPCs as an instructive signal for myelination (40). The integrin family, which links the extracellular environments of most cells with intracellular signaling molecules and the cytoskeleton via α6β1 integrin-associated protein interacting with axonal cell adhesion molecule L1 and contactin family member, as transient axonal glycoprotein (TAG)-1, has been suggested to mediate the initial axon–OL contact responsible for driving the intracellular cascade of events leading to myelination (41).

### 2.3 Myelin plasticity

In recent decades, it has become clear that myelin, far from being simply a passive insulator to ensure electric activity propagation along axons, is highly dynamic and responsive to the activity of the neurons whose axons it ensheathes (42, 43). This reflects the adaptive properties of myelin-forming cells, i.e., OPCs and OLs.

Recent results on regional heterogeneity of OPC/OL, derived from single-cell analysis, point to the possibility that two distinct modes of myelination exist: one that is independent of axonal activity and one that depends on it (34, 44, 45). Activity-dependent myelination is regulated by electrical activity and molecular cues such as growth factors, neurotransmitters, or other molecules whose expression or release is modulated by axonal electrical activity. Different putative mechanisms have been proposed (46). For example, electrical activity regulates the expression of cell surface adhesion molecules and the release of diffusible cues, such as adenosine, which inhibits OPC proliferation *in vitro* and promotes myelin formation. However, direct synaptic interactions between OPCs and unmyelinated axons may also regulate the timing of myelination. In any case, several *in vivo* studies have demonstrated activity-dependent OPC proliferation and differentiation, through exogenous stimulation of neural activity, like electrical stimulation, or endogenous stimulation of neural activity, such as voluntary exercise, and the negative effect of activity deprivation, like sensory deprivation (47). Moreover, both the absolute level of WM (i.e., the gross regions of the brain mostly populated by myelinated axons) and its rate vary throughout life, achieving their highest levels during the fifth decade of life in humans (42, 48).

In the adult CNS, myelin is fully involved in “plasticity” processes. Myelin undergoes continuous remodeling throughout life through several different processes: the addition of myelin segments, lengthening, retraction, and changes in thickness. The term “myelin plasticity” has been introduced to indicate how new sensory experiences and learning have a real-time effect on the shaping of neuronal circuits, promoting the generation of new memories and adapting CNS networks and long-distance pathways structurally and functionally to experience (49). This process guarantees the so-called experience-dependent myelination, as derived from studies linking increased oligodendrogenesis evoked by motor, spatial, and fear conditioning learning paradigms to effective learning and memory (50–52).

The occurrence and the role of experience-dependent myelination are also supported by recent evidence from studies exploring the impact of mechanical stimulation on OL biology and development. Converging scientific works report that biophysical properties of the extracellular environment, in particular mechanical cues, influence different stages of OL development, such as the differentiation of OPCs from NSCs to mature OLs (53, 54). Although the CNS mechanical niche and oligodendroglial mechanobiology require further investigation, parameters such as different mechanical cues, extracellular stiffness and topography, tensile strain, and spatial constraints tested in various *in vitro* experimental settings suggest their importance in modulating NSC differentiation toward neural or glial fates, as well as OL differentiation from OPCs (55–57), for example, reshaping the epigenetic landscape and expression of lineage and differentiation-related genes (58).

Substratum stiffness has been shown to modulate differentiation toward specific lineages, with high stiffness leading to astrocytic differentiation of NSCs, while softer substrates enhance neurogenesis and oligodendrogenesis (59). Different laboratories have reported that substrates within a defined range of stiffness increase the differentiation of rat- or mouse-derived NSCs and OPCs (54, 60). The stiffness effect is exacerbated by combining compliant substrates with chemical cues represented by specific ECM proteins, e.g., laminin or fibronectin, to achieve full OL maturation (61). Indeed, alternative ECM proteins may elicit different responses from the same cells cultured on identical substrates in terms of stiffness.

Tensile strain has also been reported to affect OL developmental processes. The main effects of strain on OLs appear to involve conformational changes of proteins, activation and opening of mechanosensitive channels induced by stretching, and chromatin reorganization through force transmission to the nucleus due to physical linking (62–64). The effects of topography and fiber geometry of substrates on NSCs and OPCs were also investigated. Topography, specifically the size and shape of physical features of a material, impacts cell cytoskeleton orientation and strength through force transmission via focal adhesion (65–67). Since myelination is also regulated by axonal diameter, electrospun nanofibers with varying diameters were tested. OPC differentiation and maturation, as well as the ensheathment of fibers with myelin, were enhanced with diameters between 2 and 4 μm (37, 68, 69). Finally, spatial constraints and compressive forces—due to both high cell density and culturing with beads—were shown to modulate and promote nuclear and epigenetic changes correlated with OL maturation and axonal myelination (58, 70, 71).

All these experimental evidences support OPCs and OLs as mechanosensitive cells and highlight the important role played by mechanical stimulation in OL biology and development, including axonal myelination. Due to its modulatory features on OL development, mechanical stimulation may be considered closely associated with concepts of myelin plasticity and experience-dependent myelination. Thus, gaining more knowledge about OPC/OL mechanotransduction pathways that lead to enhanced maturation and axonal myelination seems necessary in light of its translational implications in the field of remyelinating therapies and myelin repair.

### 2.4 Myelin repair

WM damage is currently reported in many CNS pathological conditions, such as vascular and traumatic lesions, as well as progressive neurodegenerative diseases, including Alzheimer's disease, and psychiatric conditions. Remyelination is the regenerative process by which new myelin sheaths are generated around demyelinated axons in the adult CNS. This process is usually observed in the adult CNS, and its efficiency can be very high, restoring saltatory conduction and resolving functional deficits (72–76). Remyelination is currently the only known self-repair ability of the CNS, potentially providing full anatomical and functional restoration, thus contributing to neuroprotection.

CNS remyelination is driven by OPCs that are widely distributed throughout the CNS. Usually quiescent, OPCs are activated by inflammation, which initially triggers proliferation, migration, and differentiation processes. At this stage, new OPCs can also be generated by the aNSCs in the mature CNS, such as those in the subventricular zone. The physiological OPC turnover is age-dependent, progressively decreasing with aging (77).

Adult OPCs are highly metabolically active cells and are likely the major consumers of glucose among all CNS cells. In rats, the energy consumed by WM is estimated to be about 25–50% of that consumed by GM (78), and aOPCs are responsible for a considerable share of this consumption. As a consequence, OPCs are highly vulnerable to various types of damage during adulthood, including oxygen/glucose deprivation, inflammatory cytokines, glutamate, etc. (79, 80), and their repair capability progressively declines due to cell loss and/or a block in OPC differentiation, as well described in multiple sclerosis (MS) and related animal models (74).

Remyelination is thus considered a major issue for preventing neurodegeneration and irreversible losses of function, and a therapeutic target for both acute lesions and chronic neurodegenerative diseases (81, 82).

The possibility of restoring myelin repair by stimulating endogenous OPCs is explored through various approaches, including drugs, biomaterials, exogenous cell transplantation, as well as physical activity and the delivery of physical energies. This effort is largely based on preclinical and clinical studies in MS, a condition in which remyelination has the potential to be an effective long-term strategy to both improve and protect against future disability (83). In experimental allergic encephalomyelitis (EAE), the most widely used animal model of MS, our group has extensively studied how to stimulate endogenous OPCs and NSCs to promote functional remyelination, overcoming the differentiation block of OPCs due to protracted inflammation in both laboratory rodents and non-human primates (75, 84–87). Our results have been fully confirmed by several independent laboratories in proof-of-concept studies (88), which led to a phase 1 clinical trial (NCT02760056) (89).

## 3 Rehabilitation and white matter plasticity in spinal cord injury

Basic science evidence reported in the previous pages offers a strong rationale for linking the mechanical and physical stimuli that are part of the clinical rehabilitation repertoire to myelin regeneration based on endogenous cells. MS preclinical and clinical literature offers frontline experience that can be translated to SCI. For example, ignoring the biology of remyelination could lead to poor trial design, with the consequent risk of failure in clinical trials. Many preclinical studies support the inclusion of neurorehabilitation or exercise in future MS drug trials for remyelination (90), suggesting the term “MedXercise” to describe the combination of medication and exercise to promote remyelination based on endogenous cells (91). Moreover, based on preclinical models demonstrating that aerobic exercise promotes remyelination both alone and synergistically with pharmacotherapy (92), a clinical trial to explore its translatability has been promoted, including functional (somatosensory evoked potentials) and structural (myelin water fraction) measures (93).

In the following sections, the possibility of translating/applying these efforts to SCI is discussed, focusing on specific aspects like spinal cord anatomy and imaging, and function in bipeds (humans) vs. quadrupeds (most experimental animals).

### 3.1 Spinal cord anatomy: from experimental methods to high-resolution imaging

The anatomy of the spinal cord has been described in different animal species, using anatomical and electrophysiological techniques that allow for projection labeling. Mice and rats have been widely used for anatomical studies (94), while cats have been used for electrophysiology due to their close similarity with human gray matter cytoarchitecture (95). Studies in monkeys, particularly those displaying bipedal locomotion, highlight the significant differences in neuroanatomy between rats and primates (96).

The spinal cord is composed of an H-shaped or butterfly pattern of GM, surrounded by WM formed by longitudinally running tracts that transmit information up and down the spinal cord (97, 98).

GM contains neuronal cell bodies, dendrites, interneurons, glial cells (astrocytes, microglia, and oligodendrocytes), unmyelinated axons, and synapses. It is functionally organized into 10 Rexed laminae (I-IX from dorsal to ventral), with lamina X located centrally around the central canal (the crossbar of the H). The dorsal horn, containing laminae I-VI, is the sensory part, receiving peripheral afferent signals. Axons carrying noxious and temperature impulses from peripheral receptors synapse in both lamina I and II, then cross the midline and ascend via the lateral spinothalamic tract. Lamina III and IV (the nucleus proprius) are involved in transmitting conscious proprioceptive impulses to the cerebral cortex via the dorsal medial lemniscus pathway. Lamina V receives sensory afferents from cutaneous, muscle, mechanical, and visceral nociceptors. Lamina VI, which integrates local circuits with lamina VIII, is responsible for the flexion reflex, a withdrawal response to painful stimuli. Lamina VII is present only from T1 to L2 and mainly contains autonomic motoneurons that send projections to peripheral organs and regions, including the face, neck, heart, and abdominal organs. The ventral horn, containing laminae VIII-IX, is the motor part of the spinal cord and houses the second-order motoneurons (both somatic and visceral). Lamina IX consists of a set of neuronal columns from lamina VII and VIII, innervating the muscles of the body and extremities.

WM is organized into three tracts (anterior, posterior, and lateral), where both ascending and descending pathways are arranged. There are significant differences in these pathways among different animal species, such as humans vs. non-human primates, laboratory rodents, carnivores, and other mammals.

Moreover, propriospinal fibers, referring to neurons that are intrinsic to the spinal cord and whose axons terminate within its boundaries, constitute a large proportion of the spinal cord WM and participate in various physiological and behavioral processes, including the modulation of afferent and descending input to the central pattern generators (CPG) for locomotion and respiration, as well as autonomic functions like visceroception and pain perception.

Only recently, with the introduction of high-resolution MRI for experimental animals, along with diffusion tensor imaging (DTI) and tractography, have comparative cross-species atlases been validated, and human spinal cord anatomy investigated *in vivo*, even in a longitudinal fashion after lesions (99). MRI-based 3D reconstructed models are now available for different mammal species (rat, cat, pig, monkey, and human) (100). With regard to the pyramidal tract and corticospinal tract (CST), which are heavily involved in motor control of the body (even more so in primates and humans), there is a high variability in the location of the CST among species, and tract position and size could also be subject to individual variability (99).

The GM and WM volumes of the postmortem reconstructed human spinal cord have been estimated by combining high-field MRI (9.4 T) and deep learning, found to be 2.87 and 11.33 mL, respectively, for females, and 3.55 and 19.33 mL, respectively, for males. This indicates that WM in the SC is 4 times more abundant than GM in females and 5.5 times more abundant in males (101).

### 3.2 The spinal cord injury pathophysiology in rodent models and in humans: focus on the white matter

Lesions of the CNS due to trauma or vascular accidents lead to a loss of tissue that is substituted by a non-functional scar. The pathological mechanisms causing SCI are triggered by primary injury, arising from direct spinal cord damage (traumatic, vascular, etc.). However, the functional outcome of SCI is determined by the extent of secondary degeneration, which occurs because of changes induced by the primary injury and comprises reactive, degenerative, and reparative processes. Although with different overall timing, preclinical models follow the same sequence as human neuropathology, allowing for tentative translation of cell-specific molecular signatures to phase-specific neuropathology. Phases of secondary injury are classified as the early acute phase (first 2 to 48 h, timing refers to humans), characterized by hemorrhage, edema, increasing inflammation, and a biochemical cascade of events, including free radical generation, ionic dysregulation, excitotoxicity (due to glutamate-mediated pathways), immune-related neurotoxicity, vascular disruption, axonal injury, and cellular necrosis. Preclinical data indicate that at this stage, within 15 min to 3 h after SCI, glutamate levels increase transiently, driving acute excitotoxicity. Immediately after, at 4 h a post-SCI, c-Fos and neurotransmission-related gene expression levels transiently increase, then reach their lowest peak by day 3, and go up again afterward (102). The subacute phase (day 2 to the end of week 2) is characterized by a phagocytic response to clear cellular debris, initiation of early axonal growth, edema, and necrosis of astrocytes in the area of the lesion, immune cell inflow, and scar formation that prevents axonal regeneration. The intermediate phase (week 2 to month 6) comprises maturation of the astrocytic scar and initial axonal sprouting. Finally, the chronic phase (from month 6 onward) is characterized by further scar maturation and the formation of syrinxes, ongoing Wallerian degeneration, myelomalacia, and cystic cavitations (103). These cellular and molecular signatures also drive clinical studies based on pharmaceutical intervention on druggable targets (104).

It is now recognized that the loss of WM in and around the injury site is the main cause of neurological sequelae, impairing axonal function and exposing “nude axons” to a hostile microenvironment, which results in a toxic effect also at the synaptic level (105, 106). Preclinical studies depicted the evolution of WM lesions after SCI. Immunoreactivity for CNPase, an OL marker, indicated that changes in OL occur rapidly, extending several millimeters away from the injury site. Myelin debris progressively decreased over time but could still be observed at 10 weeks after injury, especially at the more distant rostral and caudal levels from the injury site (107). OL loss precedes axonal dysfunction and loss, which are observed at the injury epicenter within 1 day of injury, peaking at 3 days post-SCI. A similar acute loss of cytoskeletal proteins was observed in rats up to 5 mm away from the injury epicenter and was particularly evident rostral to the lesion site (108). Early myelin loss was confirmed by myelin basic protein immunostaining and by electron microscopy (109).

However, while the efficiency of this process remains a matter of debate, areas of new oligodendrocytes and spontaneous remyelination were observed in all preclinical models of SCI after injury (108, 110, 111), and no persistent demyelination was found 3 months after SCI (112). More recent results describe the highly dynamic nature of the injured spinal cord over time, including persistent and concomitant demyelination and remyelination (113). When studied in T9-injured mice, the peak in remyelination occurs during the 3rd month post-injury (mpi), and myelin generation continues for at least 6 mpi. This is accompanied by changes in motor-evoked potentials, which significantly increase during peak remyelination, suggesting enhanced axonal conduction. Molecular changes, related, for example, to the ion channels expressed in the internodes, accompany this functional and anatomical dynamics. Notably, OPC processes contact glutamatergic axons in the injured spinal cord in an activity-dependent manner, and these OPC/axon contacts increased 2fold when axons were activated chemogenetically, revealing a potential therapeutic target to enhance post-SCI myelin repair.

From a clinical point of view in recent years, structural imaging based on magnetic resonance imaging, tensor-based morphometry (TBM), and diffusion imaging allowing metrics on WM integrity (such as fractional anisotropy (FA)) and tracking WM bundle orientations using DTI, provided important insights not only for clinical studies but also to validate the translational value of animal models (114, 115). These techniques offer sensitive markers of macrostructural and microstructural tissue organization, which correlate with histological findings and provide substantial evidence for the concept of WM plasticity in SCI (116).

Using structural and diffusion-weighted MRI, the spatiotemporal dynamics of tissue-specific spinal cord neurodegeneration above and below a spinal cord injury have been described in patients. Longitudinal studies indicate that, in humans, WM atrophy precedes GM degeneration, at least above the lesion level (117). These studies also provided evidence for long-distance WM changes, as seen in the cerebral cortex (118), and in many WM tracts, as indicated by decreased FA and increased mean diffusivity and radial diffusivity in the corpus callosum, superior longitudinal fasciculus, corona radiata, posterior thalamic radiation, right cingulum, and right superior fronto-occipital fasciculus (119).

### 3.3 Remyelination improvement by physical energies: preclinical evidence

Due to the impact of neural activity on developmental myelination and myelin plasticity previously discussed, and since remyelination at least partially recapitulates developmental myelination, the question here is whether neural activity could also impact remyelination. Evidence from animal models using experimental tools for neural activation (chemogenetic and optogenetic approaches) suggests that stimulation of demyelinated fibers is able to promote OPC differentiation and remyelination (120).

This also seems to be true when using clinically relevant stimulation, as neuronal activity induced through epidural electrodes implanted over the primary motor cortex increased the number of proliferating OPCs, the number of oligodendrocytes, enhanced MBP expression, and myelin sheath formation, ultimately promoting the recovery of hindlimb motor function (121). Transcranial low-frequency pulsed electromagnetic fields demonstrated enhanced remyelination through increased oligodendrogenesis and attenuation of inflammation, an effect that seems to be mediated by the release of BDNF and NGF by stimulated axons (122). NGF also protects OPCs from a hypoxia-generated microenvironment (123). Transcranial direct current stimulation (tDCS) also improves remyelination by enhancing the effects of cell therapy (124). Transcranial focused ultrasound seems to be able to increase myelin regeneration in cuprizone-fed demyelinated mice (125).

Moreover, different techniques of non-invasive brain stimulation have been demonstrated to slow down myelin breakdown in the demyelination phase (126–128). OPC-induced myelin repair through stimulation of axonal propagation promotes motor function recovery in SCI (129). Mechanical cues also seem to have an effect in promoting neural repair and remyelination. Mechanical stimulation, such as tensile loading or scaffolds mimicking the mechanical properties of the original tissue environment, has been used to promote neural regeneration and functional recovery in various conditions. Tensile loading and neural mobilization have mostly been investigated in the context of nerve repair after PNS injury as a neurodynamic therapeutic technique. *In vitro, in vivo*, and clinical trial evidence supports the tensile loading approach as an enhancer of the remyelination process (130). A deeper investigation of this strategy is required, particularly regarding CNS stimulation after injury. Scaffold-based approaches aim to mimic the mechanical and topographic properties of the original ECM to promote axonal growth, regeneration, and remyelination. Indeed, specific substrate stiffness and topography seem to play important roles in promoting OPC differentiation and myelin sheath formation in both *in vitro* and *in vivo* models (131, 132). For instance, Liu et al. reported that 3D-printed BDNF/collagen/chitosan-based scaffolds transplanted in a rat injury model promoted spinal cord regeneration, synapse establishment and remyelination at the injury site (133). The responses of OPCs to mechanical stimulation and the activated pathways still need to be further elucidated; nevertheless, evidence supports mechanical stimulation as an important biophysical factor to consider when designing therapeutic strategies for axon remyelination after injury, along with all the aforementioned approaches.

In addition to exogenous stimulations, exercise-induced neural activity has also been demonstrated to be effective in myelin repair in animal models (134, 135). Treadmill training has been shown to enhance remyelination and subsequent functional performance and potential conduction after SCI by promoting OPC proliferation and OL maturation, increasing MBP expression and myelin sheath thickness through the upregulation of oligodendroglial peroxisome proliferator-activated receptor γ coactivator 1α (PGC1α) (135).

Although still limited, experimental programs for animal training include quadrupedal and bipedal treadmill training, cycling, swimming, and climbing training, eventually supported by robotic assistance; all protocols are adapted to avoid unfavorable training conditions that could lead to hypersensitivity (136). For example, rehabilitation training involving skilled reaching and grasping tasks improves performance after injury (137).

Also, combinatory treatments have been tested, such as simultaneous combined treadmill training and magnetic stimulation, that significantly improved spasticity and gait impairments after cervical SCI. The decreases in the deficiency density ratio of spared WM are indicated among the possible mechanisms, along with the upregulation of dopamine beta-hydroxylase, glutamic acid decarboxylase, gamma-aminobutyric acid receptor B, and brain-derived neurotrophic factor compared to untreated animals (138). In addition, another study found that low-intensity repetitive transcranial magnetic stimulation (TMS) increased the number of newborn oligodendrocytes in the adult mouse cortex by increasing cell survival and improving myelination (139).

Notably, more data are now available and accessible for further analysis, dealing, for example, with spatial multi-omics technologies in single-cell populations (140), or more specific studies, such as the transcriptome of rat subcortical WM and spinal cord after spinal injury and cortical stimulation (141).

Regenerative rehabilitation studies in preclinical models of spinal cord injury, including the chronic phase, have been recently reviewed (142). For the purposes of this article, we highlight the effects of different rehabilitation training paradigms on microstructure around, but also far from, the lesion, which includes increased myelin protection and repair of both ascending and descending tracts and restoration of motor-evoked potential amplitude.

### 3.4 Remyelination improvement by rehabilitation: clinical evidence

Considering the emerging role of WM plasticity and repair in functional recovery from SCI, rehabilitation programs could also be adapted to foster remyelination, as suggested by studies in MS patients that support the use of neuromodulation and rehabilitation exercises, along with non-pharmacological strategies for promoting remyelination (143).

Given the many existing rehabilitative therapies and approaches, various stimuli can be applied. Several studies show that conventional rehabilitation, which mainly consists of motor skills training, stimulates remyelination by applying mechanical stimuli. There is also increasing evidence that physical stimuli, such as electrical and magnetic stimulation, can promote neuroregeneration by enhancing, inhibiting, or regulating neural cell activity. In addition, considering the progress in the field of medical engineering technology, some new rehabilitation techniques, such as robotic rehabilitation, have rapidly developed and are now widely used in neurorehabilitation, demonstrating that even robot-assisted training can increase neuroplasticity by providing different kinds of stimuli.

Although several preclinical studies have attempted to assess the synergistic effect of remyelination and rehabilitation, very few clinical studies have investigated this combinatorial effect in human SCI patients. Recent advances in neural imaging have made it possible to demonstrate rehabilitation-induced structural changes in both gray and white matter.

In clinical practice, conventional rehabilitation mainly consists of physical exercise and motor skills training, such as balance, hand and reaching function, transfer, and gait (136). These joint range-of-motion exercises and muscle strengthening provide mechanical stimuli to the cells.

Several studies have already shown that physical exercise induces white matter plasticity in physically healthy humans. In particular, by using proper neural imaging, it is possible to track and quantify training-associated remyelination in different tracts of the spinal cord, as well as changes in white matter brain structure assessing the effects of interventions (144). Similar results have been obtained when the effects of rehabilitation on white matter plasticity have been investigated in patients with SCI: myelin water imaging revealed increased myelin water fraction (MWF), an imaging biomarker of white matter, in brain motor learning regions and mixed motor and sensory tracts of the ventral cervical spinal cord after motor skill rehabilitation training in humans with SCI (134).

Physical stimuli are also included in the routine repertoire of clinical rehabilitation. Transcranial magnetic stimulation, transcutaneous spinal direct current stimulation (tsDCS), and transcranial direct current stimulation are non-invasive stimulation techniques used as therapeutic strategies to improve the functionality of patients with SCI (145–147). In clinical practice, these techniques have recently been introduced, often in combination with different exercise strategies like locomotor training, and they depend on the protocol for using the technique (148). TMS can be used to stimulate specific CNS tissues, affect local cell activity and increase corticospinal tract excitability by transmitting magnetic pulses from copper coils (149).

tsDCS delivers weak direct current (1–5 mA of intensity; 0.027 to 2.3 mAh/cm^2^ of charge density) through a pair of skin electrodes to modulate conduction along spinal somatosensory pathways via an induced electric field. A study involving animal subjects has provided evidence that non-invasive tsDCS can facilitate corticospinal drive for one muscle preferentially over another, depending on electrode location (150). With regard to tDCS, a study in adults with chronic incomplete cervical SCI that evaluated the effect of active tDCS treatment vs. sham treatment followed by 1 h of robot-assisted arm training found a positive trend in DTI, demonstrated as an overall increase in the FA change of corticospinal tracts (151).

Due to the development of medical biology and medical engineering technologies, new approaches to rehabilitation are growing rapidly, with the field of robotic rehabilitation being one of them. Robotic devices and all types of body–machine interfaces, which translate signals derived from body movements into commands for external devices and return real-time feedback, provide a wide range of multimodal stimuli that integrate visual, auditory, spatial, and proprioceptive information (152).

Recent clinical studies have shown that robot-assisted rehabilitation training increases neural imaging parameters associated with myelin formation in both SCI and healthy patients (151–154). These results may also be related to the fact that robotic rehabilitation increases patient engagement and participation through the use of virtual reality, gaming, and technology-assisted interaction. The level of engagement during rehabilitation has a significant impact on active participation, which stimulates neural plasticity: the more involved the patient is, the more CNS plasticity is promoted (155, 156).

## 4 Bench-to-bed and bed-to-bench: possible good reciprocal news

There is increasing evidence from both preclinical and clinical literature that rehabilitative treatments promote myelin repair through the mobilization and differentiation of endogenous OPCs into myelinating oligodendrocytes, inducing WM changes that promote CNS adaptation to different functions and improve functional recovery in people with neurological disorders such as SCI. While there is substantial literature in preclinical models supporting these findings, there are still few clinical trials investigating these issues. However, many of the results obtained in preclinical studies can be extended to the clinical setting to fill this gap, particularly by using the latest imaging techniques that can monitor WM lesion evolution and myelin repair.

**Figure 1:**
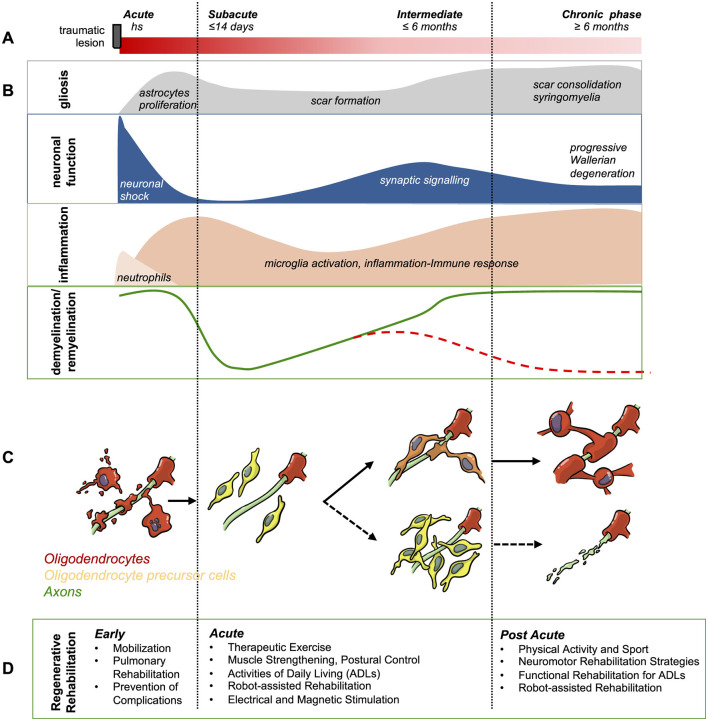
Timeline of the main pathological events during secondary degeneration after spinal cord injury and possible regenerative rehabilitation approaches. **(A)** Indicative timing of early, subacute, intermediate and chronic phase. **(B)** Profile of the main pathological events characterizing secondary degeneration after traumatic injury. Inflammation, characterized by the invasion of damaged nervous tissue by peripheral white blood cells, activation of microglia, and further recruitment of lymphocytes, is the main trigger of the astroglial reaction, which leads to scar formation. Neuronal function is marked by an early functional shock, followed by the re-emergence of synaptic signaling by residual terminals. Myelin is severely damaged, and its repair could be substantial (green line) or null (red line), depending on the behavior of OPCs. **(C)** The inflammatory microenvironment and excess glutamate contribute to the secondary degeneration of OL, leaving highly vulnerable “nude axons.” In the meantime, inflammatory cytokines stimulate the proliferation of OPCs, whose fate is strongly influenced by the microenvironment. If inflammation is controlled and axons release appropriate signaling molecules, OPCs approach nude axons and remyelinate them. If inflammation is not controlled and/or axons are silent and not releasing attracting molecules, OPCs fail to differentiate into myelinating OL, leading to the progressive degeneration of nude axons and long-distance Wallerian degeneration. **(D)** Possible rehabilitation interventions promoting endogenous regeneration, tailored to the different post-lesion phases.

We summarized in Figure 1 the time course of the main pathological events in the spinal cord after SCI (neuronal function, inflammation, scar formation, demyelination/remyelination), OL/OPC biology, and the potential impact of regenerative rehabilitation approaches, optimized according to the clinical phase and the patient's conditions. The state of knowledge regarding SCI pathophysiology and the underlying biological mechanisms derived from preclinical studies has allowed for a more specific definition of possible strategies and timing of interventions, considering both rehabilitation and pharmaceutical approaches. Some studies have shown that the best period to maximize the WM changes induced by treatments occurs between the 3rd month and 6th month post-injury, corresponding to the peak of remyelination. Others have demonstrated that common rehabilitation exercises, such as treadmill training, promote oligodendrogenesis and WM plasticity even in the chronic phase. In addition, *in vivo* studies have shown both activity-dependent proliferation and differentiation of OPCs through various types of stimuli and highlighted the negative effects of activity deprivation, underscoring the importance of minimizing inactivity and sensory deprivation. These findings can be applied clinically to target the best interventions at the right time; for example, an early start of rehabilitation treatment could be proposed to create the optimal environment for remyelination and protection against neurodegeneration during the critical window of the first six months post-injury, followed by long-term continuation with general exercise activities, such as sports, to promote WM plasticity and, consequently, continuous improvement of functional motor performance. More generally, it would be desirable for specific endpoints aimed at investigating WM repair through appropriate MRI techniques and analysis, including tractography, as well as axonal function through electrophysiology, to be included in the study design of future rehabilitation trials in SCI.

Moreover, future studies should investigate the effects of multimodal interventions that combine rehabilitation treatments with pharmacological therapies, following the increasingly relevant and necessary perspective of personalized treatment tailored to inter-individual variability.
